# Providing pediatric well-care and sick visits in the COVID-19 pandemic era: the recommendations of the Italian pediatric society

**DOI:** 10.1186/s13052-020-00899-0

**Published:** 2020-09-16

**Authors:** Rino Agostiniani, Elena Bozzola, Annamaria Staiano, Antonio Del Vecchio, Teresa Mazzone, Luigi Greco, Giovanni Corsello, Alberto Villani

**Affiliations:** The Italian Pediatric Society, Rome, Italy

**Keywords:** Children, COVID19, Visit

## Abstract

Pediatricians have observed a significant decrease in in-person child health visits during the COVID-19 pandemic. In the post lockdown period, the coronavirus trend remains positive in Italy but fears of a second wave have recently grown in Italy due to active hotbeds of contagion. The pandemic may negatively affect the care of pediatric patients and overall children welfare as it may present with severe signs and symptoms or it may complicate. The Italian Pediatric Society recommend to separate well visits from sick ones, to educate families and to promote hygienic strategies to provide an adequate pediatric assistance in case of a second pandemic wave.

## Background

Pediatric patients with coronavirus disease 2019 (COVID-19) may experience flu-like signs or symptoms over the course of the disease, such as fever, cough, nasal congestion or rhinorrhea, sore throat, diarrhea, nausea or vomiting [1, 2].

In children illness severity may range from an asymptomatic to a critical condition. Clinical presentation may be as follow [1]:

- asymptomatic
- mild (including fever, fatigue, myalgia, cough)
- moderate (such as pneumonia)
- severe (such as dyspnea, hypoxia)
- critical (such as acute respiratory distress syndrome, respiratory failure, shock, or multi-organ dysfunction).

In Italy, the pediatric cases are actually 2100 in children aged 0–9 years and 3745 in those aged 10–19 years, respectively 0,9% and 1,6% of the total cases [3].

Fewer cases of COVID-19 among children compared to adult cases are reported, partially due to under diagnosis [4]. In fact, most pediatric cases are asymptomatic or with mild-moderate symptoms [5]. Nevertheless, pediatric cases of COVID-19, caused by severe acute respiratory syndrome coronavirus 2 (SARS-CoV-2), have been reported, as well as rare hyperinflammatory states (Kawasaki-like syndrome). Due to the recent onset of COVID-19 pandemic disease, long term consequences are inevitably unknown at present. Data suggest that toddler younger than 12 months as well as underlying medical conditions represent severity risk factors [5].

Age-stratified analysis showed that the SARS in children was 4.7% compared with 17.1% in adults and that the odds of infection in children was 0.26 times (95%CI 0.13–0.54) of that among the elderly [6, 7]. Although most pediatric cases are mild, pediatricians should maintain suspicion for SARS-CoV-2 infection in children, visit sick patients and monitor their clinical conditions for the risk of complications, in order to avoid the spread of the infection to other children, schoolmates caregivers, family members and physicians.

In the post lockdown period, the coronavirus trend remains positive in Italy but there are still active hotbeds of contagion**,** according to the monitoring report by the Health Ministry and the Health Institute (ISS). These are small but they are also sufficient for the experts to say that the COVID-19 epidemic in Italy is not definitely over yet and that it is still necessary to keep the guard up and maintain social-distancing measures.

Some countries have experienced a second wave of cases, which consisted in a rise in infections after appearing to have the virus under control, such as Israel and Spain.

Fears of a second wave have recently grown in Italy, relating next fall/winter season.

## The Italian pediatric society recommendations

Pediatricians have observed a significant decrease in in-person child health visits during the COVID-19 pandemic, which puts children’s health at risk.

Moreover many parents have concerns about what to do if their child will be sick in the next months.

The Italian Pediatric Society suggests parent to contact the child’s pediatrician/physician if they have concerns. It is important at this time to not immediately assume an emergency room visit if it is unneeded. On the contrary, in case of necessity, discourage families remaining at home for the fear of infection.

As well as the American Academy of Pediatrics, the Italian Pediatric Society strongly supports the continued provision of health care for children [8].

On March 24, CDC posted guidance emphasizing the importance of routine well child care and immunization, particularly for children aged ≤24 months [9]. The current emergency might negatively affect the care of pediatric patients and overall children welfare. In particular, the fear of contracting COVID-19 may determine a delayed access to pediatric emergency facilities as pointed out in a recent study. Moreover, as parents are discouraged from entering hospitals even in case of severe clinical conditions, children were more frequently admitted in compromised situations [10].

Regarding healthcare providers, as COVID 19 is not yet over in Italy, the Italian Pediatric Society recommend to use strategies to separate well visits from sick ones. A suggestion may be to schedule routine appointments in the morning and sick visits in the afternoon. Sanitation and hygiene services are an essential part of preventing and protecting people during infection outbreaks, such as the one caused by COVID 19. Promoting good handwashing behavior, hygiene, physical distancing and the use of face masks as the most effective strategies to prevent infection may part of daily communication activity to families.

In the latest months, a significant drop in well-child visits has resulted in delays in vaccinations, delays in appropriate screenings. In some cases, pediatricians have rapidly adapted to assist children through telehealth. However, in most cases, a in-person visit is required to perform a physical exam.

Scheduling sick visits and well-child visits during different times of the day and/or different locations should help families not to miss appointments. Physical and neurodevelopment screening should continue in order to provide both an early intervention service and a prompt assistance in case of pathological findings. In the meantime, healthcare providers should identify children who have missed routine controls and/or vaccinations and contact them to schedule in person appointments.

Even in pandemic, all newborns should be seen by a pediatric healthcare provider shortly after hospital discharge and should be evaluated for peri-natal problems such as jaundice, loss of weight, etc. ..

As for routine children check, the Italian Pediatric Society recommends:

- schedule appointments and fill a telephonic family interview to find out risks factors (for example exposure to and symptoms consistent with the coronavirus) as reported in Table 1.
- reducing crowding in waiting rooms, planning appointment and asking patients to remain outside the medical office until they are called into the room for their appointment
- upon entering medical room, check temperature to the child and to the caregiver. Both the patient, according to age, and the caregiver should wear a surgical mask and adopt hygienic practices (such as washing hands)
- inform families about the strategies already implemented in medical home office to assure safety.
- educate families and provide the information to avoid the spread of infections, by everyday preventive actions, as reported by WHO [11].
- explain health precautions put in place to help ensure the safety of the children during medical appointment (such as use facial mask and wash your hands before and after the medical visit)

**Table 1:** Telephonic COVID screening

a) Has the child or a cohabitant recently travelled? ^a^	
b) Has the child or a cohabitant have been in contact with a confirmed case of coronavirus?	
c) Has the child or a cohabitant have been in contact with someone returning from a moderate/high risk countries or places? ^a^	
d) Has the child fever or flu-like symptoms (such as cold, cough, vomiting or diarreha, headache or malaise)?	
e) Has the caregiver or a cohabitant flu-like symptoms?	
f) Has the caregiver or a cohabitant smell and/or taste loss?	
g) Has the caregiver or a cohabitant presented flu-like symptoms in the last 30 days?	
h) Has the caregiver or a cohabitant presented smell and/or taste loss in the previous 30 days?	

^a^Consider moderate and high risk area according to COVID 19 changing epidemiology

In case of sick children, the Italian Pediatric Society recommends to identify those with signs or symptoms compatible with COVID-19 and:

- schedule different appointments, separated from COVID 19 unrelated flu like patients.
- reducing crowding in waiting rooms, by asking patients to remain outside the medical office until they are called into the room for their appointment. It should be advisable to have just one caregiver with the patient inside the medical room.
- ask the patient, according to the age, and the caregiver to wear a facial mask and adopt hygienic practices (such as washing hands)
- discourage admission to hospital in case of mild symptoms like fever or viral symptoms unless severity or no other alternative arrangements can be made
- educate families and provide information to avoid the spread of infections
- in case of suspected COVID-19, healthcare providers should use specific personal and protective equipment rather than a facial mask [12].
- consult official flow diagram for the management of ill children with suspected COVID-19 infection provided by the Health Ministry.

## Discussion

Pandemics implicates many worries in the various settings of pediatrics, including primary, secondary and tertiary care. A major problems is that children are possibly less sick than adults, but most children live in large communities not capable to self management, including primary needs. In the latest years, people are searching for health online. A recent survey found out that more than half of the respondents (54%) used the search engine at least weekly to look up medical questions and symptoms and more than 40% use Google as the only source of information on health. The results may have important repercussions on people’ health, given that most of the time, “Dr Google” doesn’t provide the right diagnosis [13].

Pediatricians should continue providing routine preventive and other nonemergency care, discussing with caregivers on the benefit of attending a in-person visit, immunizations and screenings to avoid both missing visits and wrong diagnosis trusting on-line information.

## Data Availability

At Bambino Gesù Children Hospital, Dr. Bozzola’s repository
